# Natural toll-like receptor agonists in prophylactic vaccines for dendritic cell activation

**DOI:** 10.1186/2051-1426-3-S2-P446

**Published:** 2015-11-04

**Authors:** Paul Peng, Linda Hammerich, Joshua Brody

**Affiliations:** 1 Icahn School of Medicine at Mount Sinai, New York, NY, USA

## Introduction

There is an unmet need for novel and effective treatments for lymphoma, the fifth most common malignancy in the US. *In situ* vaccination—an immunotherapeutic maneuver involving local irradiation, intratumoral (i.t.) injections of Flt3L and Toll-like receptor (TLR) agonist—has been shown in recent clinical trials (NCT00185965, NCT00880581, NCT00226993) to induce partial and complete remissions in patients with low-grade lymphoma[1]. The strength of anti-tumor response correlates with the potency of immunogenic dendritic cells (DCs) to efficiently uptake and present tumor antigens to T cells[2]. While the latest clinical trial NCT01976585 employs Poly-ICLC—a synthetic TLR3 agonist—to activate DCs, we hypothesize that “natural” TLR agonists (nTLRa) contained within prophylactic vaccines could simultaneously target multiple TLRs and be repurposed as clinical-grade DC activators for the *in situ* vaccination maneuver[3].

## Methods

Twenty-four prophylactic vaccines were screened for TLR ligand activity using *in vitro* assays. DC subsets CD11c^+^MHC-II^+^, CD11c^+^CD11b^+^, and CD11c^+^CD103^+^ were gated for: (i.) activation markers (e.g. CD80, CD86, MHC I, II), (ii.) ability to co-stimulate T cells in the context of TCR activation, as assessed by T cell activation markers (e.g. CD69, intracellular IFNγ), and (iii.) ability of nTLRa-activated DCs to induce T cell proliferation. TLR knock-out cell lines were used for dissecting the mechanistically-distinct properties of each prophylactic vaccine. Best single and combination nTLRa candidates were evaluated with the *in situ* vaccination maneuver in an *in vivo* A20 murine lymphoma model.

## Results

**Figure 1:**
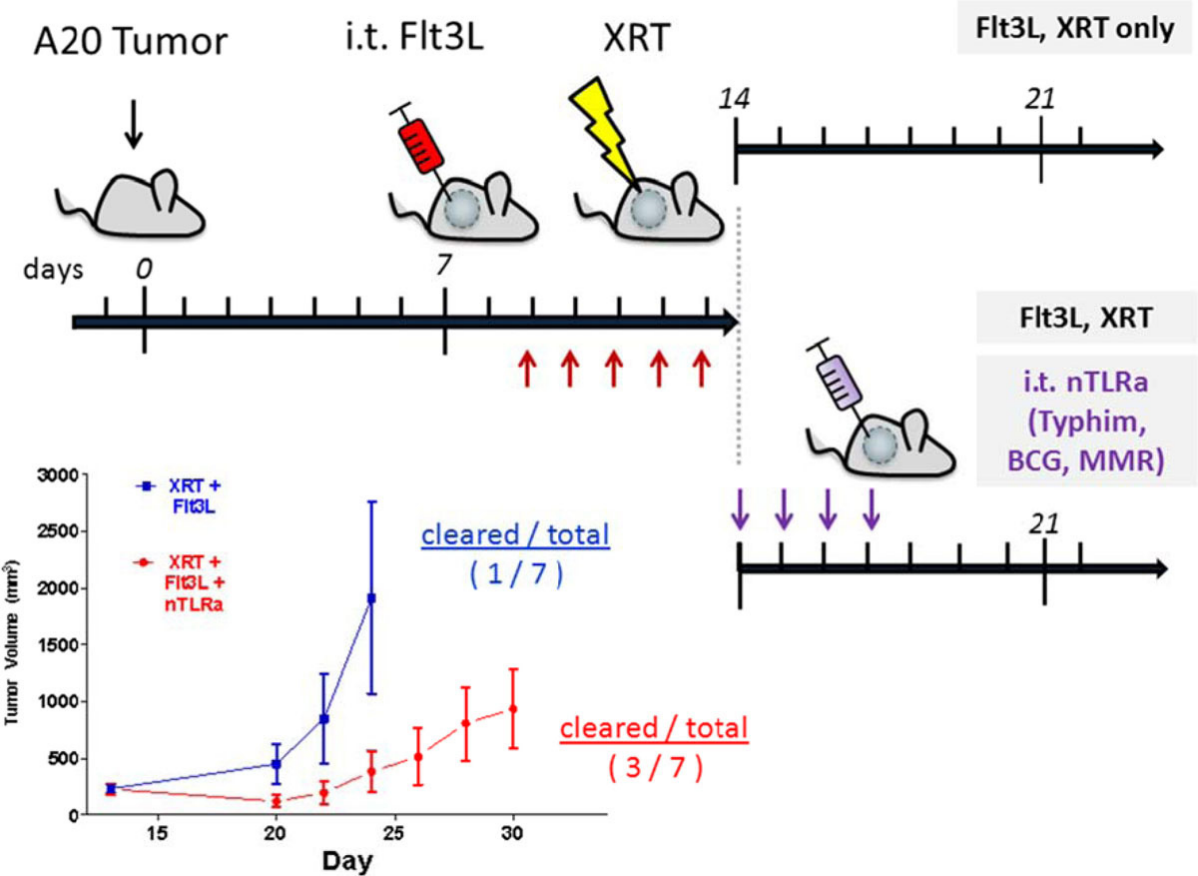


**Figure 2:**
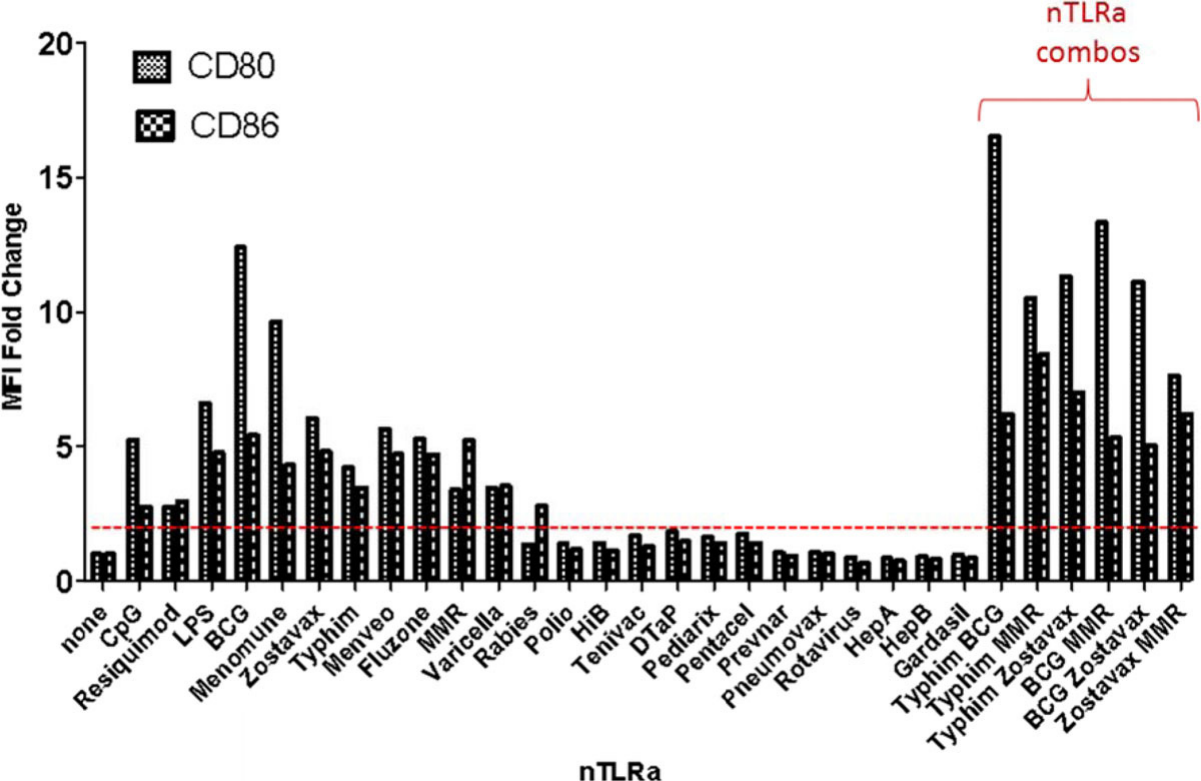


We identify a combination of vaccines Typhim, BCG-TICE, and MMR that possess the ability to induce high expression of costimulatory molecules on DCs *in vitro* (Figure 1), as well as co-stimulating *in vitro* T cell activation and proliferation. DC costimulation of T cell activation and proliferation is highly dependent on the “live” status of several vaccines, including BCG, MMR, Zostavax, suggesting the involvement of other pattern-recognition receptors in addition to TLRs. Cohorts of mice implanted with A20 lymphoma tumors that received i.t. injections of Typhim, BCG, MMR demonstrated slower tumor growth status as compared to the control cohort receiving only Flt3L and local irradiation (Figure 2).

## Conclusions

Prophylactic vaccines contain natural ligands to TLRs and are immediately translatable as sources of clinical-grade stimulators of dendritic cells. Combinations of the best nTLRa candidates display synergistic activation of DCs and *in vivo* anti-A20 murine lymphoma immunity.
